# Stop codon readthrough contexts influence reporter expression differentially depending on the presence of an IRES

**DOI:** 10.12688/wellcomeopenres.16231.2

**Published:** 2021-10-06

**Authors:** Martina M. Yordanova, Gary Loughran, John F. Atkins, Pavel V. Baranov

**Affiliations:** 1 School of Biochemistry and Cell Biology, University College Cork, Cork, T12 XF62, Ireland; 2 Shemyakin and Ovchinnikov Institute of Bioorganic Chemistry, RAS, Moscow, Russian Federation

**Keywords:** Translation control, AMD1, stop codon readthrough, IRES, OPRL1, ribosome stalling

## Abstract

**Background:** Previously we reported the discovery of stop codon readthrough in *AMD1* mRNA followed by ribosome stalling at the end of a conserved Open Reading Frame (ORF) that we termed *AMD1*. To explain the severe suppression of reporters fused to *AMD1* tail we proposed a mechanism invoking ribosome queueing. To test this hypothesis, we placed the reporter stop codon in the context of readthrough permissive sequences in a dual reporter vector with downstream reporter expression driven by the EMCV IRES. In accordance with our hypothesis, we observed a striking disproportional reduction of upstream reporter activity in response to increased readthrough levels.

**Methods: **We employ dual luciferase assays, western blotting and RT-qPCR to explore the effects of test sequences downstream to the reporter stop codon on its expression in dual and monocistronic reporter vectors.

**Results:**  With the dual reporter system, the disproportionate reduction of upstream reporter activity is not specific to *AMD1* tail and occurs as long as the readthrough stop codon context is present at the end of the reporter’s ORF. In a monocistronic vector without an IRES, the test sequences had distinct effects which were reflective of their properties e.g. *AMD1* tail inhibitory effect. We further show with RT-qPCR that the EMCV IRES driven expression of a reporter is an accurate proxy of reporter RNA levels.

**Conclusions:** While our findings provide little new information regarding the functional role of *AMD1* tail, they raise caution for the use of viral IRES elements in expression vectors for studying mechanisms of mRNA translation. These findings may also be pertinent to the natural properties of read through permissive sequences and of IRES elements, though these require a separate investigation.

## Introduction

Recently we discovered that a proportion of ribosomes translating the *AMD1* mRNA read through its annotated stop codon and continue translating before stalling at the end of a 125-codon conserved open reading frame (ORF), referred to as *AMD1 tail* ( Yordanova *et al*., 2018). We proposed that ribosome stalling leads to queueing that can inhibit translation of the *AMD1* coding sequence. Ribosome stalling at the end of *AMD1 tail* and its dependence on stop codon readthrough (RT) has since been confirmed in a more recent study ( Wangen & Green, 2020). During our initial investigation of this phenomenon, we found that fusing the product of *AMD1 tail* translation, i.e. *AMD1* extension, to the C-terminus of reporters leads to nearly complete disappearance of reporter activity ( Yordanova *et al*., 2018). After ruling out extracellular targeting or a protein destabilisation effect of the *AMD1* extension, we proposed a mechanism where ribosome stalling/queuing at the end of *AMD1 tail* results in inhibition of the upstream main ORF translation. A prediction of this mechanism is that increasing the readthrough efficiency at the main ORF stop codon should accelerate the *AMD1 tail* inhibitory effect by enhancing queue formation. We tested this prediction with RT promoting sequences of varying efficiencies from *LDHB*, *AQP4* and *OPRL1* genes ( Loughran *et al*., 2014; Loughran *et al*., 2017) to titrate ribosomes translating the *AMD1 tail* ( Yordanova *et al*., 2018). By increasing the RT efficiency at a reporter’s stop codon to 2.5, 6 and 17% with *LDHB*, *AQP4* and *OPRL1* contexts, respectively, we observed a disproportionately large drop of reporter levels, i.e. beyond what would be expected due to protein degradation if AMD1 extension had a destabilization effect as proposed for other products of 3’UTRs translation ( Arribere *et al*., 2016).

In a follow up examination, we applied the above-described approach of RT-enabled ribosome titration to further explore the dynamics of *AMD1 tail* translation and that of other test sequences. These experiments, as in the original study, were performed with a bicistronic dual luciferase vector wherein the termination codon of the Renilla luciferase (Rluc) reporter was placed in a RT permissive context just upstream of the test sequence. A firefly luciferase (Fluc) reporter was expressed *via* an EMCV IRES ( Chamond *et al*., 2014) to monitor RNA levels and to control for varying transfection efficiencies. We show here that the inhibitory effect on the upstream reporter is not mediated by the test sequence but rather depends on the presence of the RT signal and is specific to the IRES harbouring vector.

## Methods

### Cloning

Oligonucleotides were synthesized by IDT, Belgium. *AMD1* tail, *ODC1* PEST and *ACTB* sequences were obtained as gBlocks from IDT. gBlock and primer sequences including those that introduce *OPRL1*, *AQP4* and *LDHB* stop codon context sequences are provided in *Extended data* File 1 ( Yordanova *et al.*, 2020b). The amplicons were generated by standard one-step or multiple-step PCR using Phusion High Fidelity DNA Polymerase (NEB) according to the manufacturer instructions. p2luc ( Grentzmann *et al*., 1998) was modified such that the second luciferase reporter (Fluc) is expressed under the control of the EMCV IRES. Due to the presence of an XbaI restriction site in *AMD1 tail*, the first 65 nts of *AMD1 tail* were omitted for cloning in the monocistronic vector. All constructs were transformed by 90 sec heat shock at 42°C in *E. coli* strain DH5-α and were verified by Sanger sequencing at Eurofins Genomics.

### Tissue culture and cell treatment

Human Embryonic Kidney 293A cells (ATCC) were maintained as monolayer cultures, grown in DMEM (Sigma-Aldrich) supplemented with 10% FBS, 1mM L-glutamine and 1% penicillin/streptomycin at 37°C in an atmosphere of 5% CO _2_. For dual luciferase assays 4.5×10 ^6^ HEK293A cells were plated on 10 cm tissue culture dishes. After 24 h the cells were detached with trypsin, suspended in fresh media and transfected in four replicates with Lipofectamine 2000 reagent (Invitrogen), using the 1-day protocol in which suspended cells are added directly to the DNA complexes in 96-well plates. For each transfection, the following was added to each well: 25 ng plasmid DNA and 0.2 μl lipofectamine 2000 in 25 μl OptiMem (Gibco). 2×10 ^4^ cells in 50 μl DMEM, were added to the transfecting DNA complexes in each well. Transfected cells were incubated at 37°C in 5% CO _2_ for 21 h and assayed using the dual luciferase assay. Data shown on the figures were obtained from three independent transfections each with four technical replicates.

### Dual luciferase assay

Fluc and Rluc assay buffers were prepared as described in ( Dyer *et al*., 2000). Relative light units were measured on a Veritas Microplate Luminometer fitted with two injectors (Turner Biosystems). Cells transfected in 96 well plate were washed once with 1× PBS and then lysed in 15 μl of 1× passive lysis buffer (PLB; Promega). Light emission was measured following injection of 50 μl of each luciferase substrate buffer. Raw data for the dual luciferase assays are available as Underlying data ( Yordanova *et al*., 2020a).

### Protein isolation and western blot analysis

**Figure 1.**
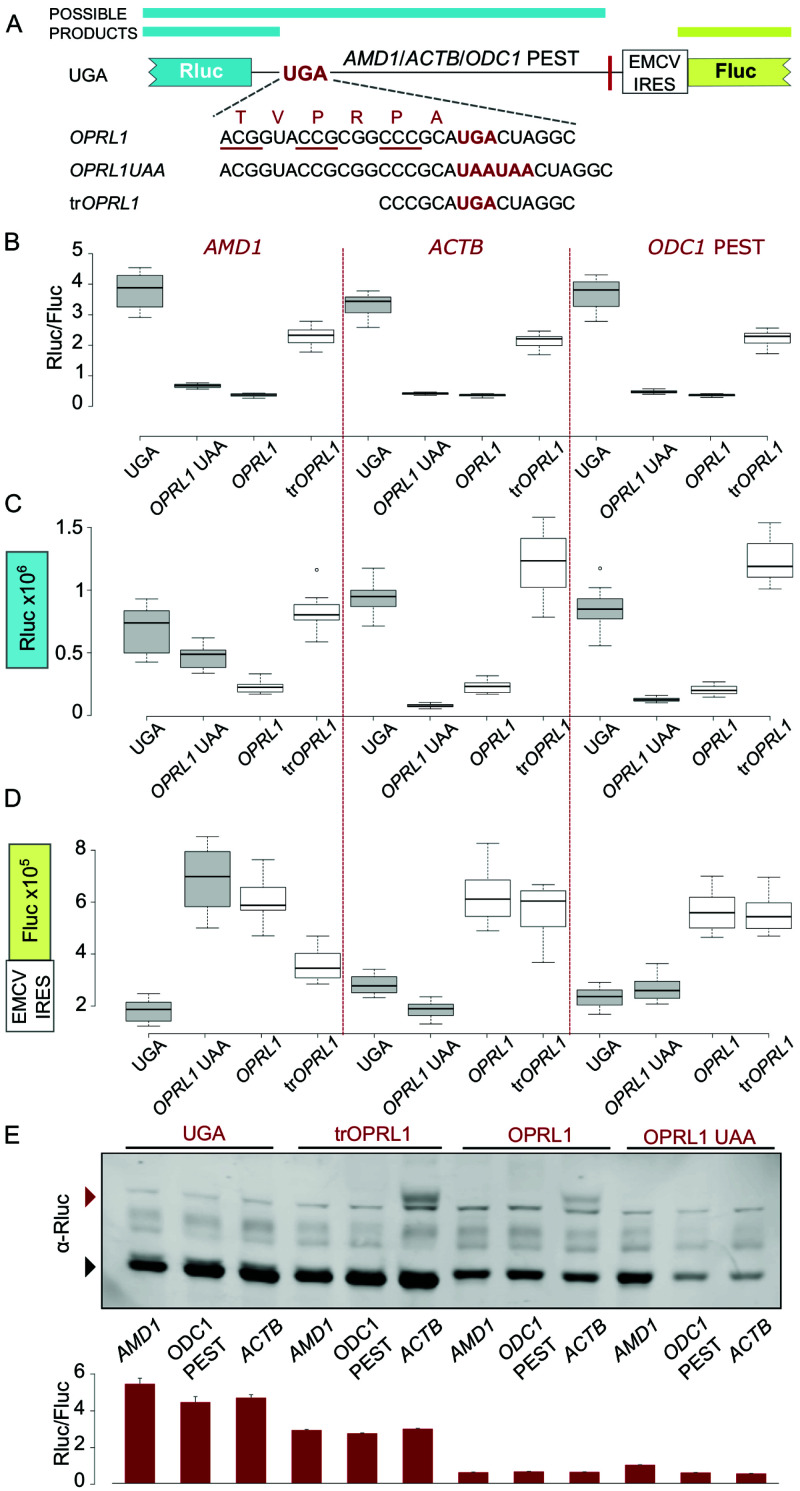
*OPRL1* readthrough context mediated inhibition of Rluc levels in EMCV IRES vector. ( **A**) Schematic of the *AMD1*, *ACTB* and *ODC1* PEST constructs where Rluc stop codon context is varied and Fluc expression is governed by the EMCV IRES. ( **B**) Normalized (Rluc/Fluc) activities. ( **C**) Absolute Rluc values. ( **D**) Absolute Fluc values. ( **E**) Upper panel, Anti-Rluc immunoblots of protein lysates from HEK293A cells transfected with the indicated constructs; termination products are indicated with a black arrowhead, readthrough products (seen only in *ACTB* constructs) are indicated with a red arrowhead; lower panel, normalised (Rluc/Fluc) activities from the protein lysates. See Methods for box plot elements.

Transfections for Western blotting analysis of constructs for Figure 1 were performed in 6 well plates scaled-up from the method described for 96 well plate transfections above. The following was added to each well: 1 µg plasmid DNA, 7 μl lipofectamine 2000 in 1 ml OptiMem. A total of 1×10 ^6^ cells in 3 ml DMEM, were added to the transfecting DNA complexes in each well. Transfected cells were incubated at 37°C in 5% CO _2_ for 36 h for Western blotting. Cells were washed with 1x PBS and lysed in 1x PLB (Passive Lysis Buffer, Promega). Luciferase activities in the lysates were measured with the dual luciferase assay. Proteins were separated by 4–12% polyacrylamide gel electrophoresis on precast BoltTM 4–12% BisTris Plus gels (Thermo Fisher), transferred onto nitrocellulose membranes (Protran) and incubated with primary Rabbit Anti-Renilla Luciferase Polyclonal Antibody (1000x dilution) (MBL International) (RRID: AB_1520866) in 5% fat-free milk in PBST (1% Tween-20) overnight at 4°C. Incubation with IRDye® 800CW Goat anti-Rabbit IgG Secondary Antibody (10,000x dilution) (Abcam, ab216772) was for 0.5 h at room temperature.

### RT-qPCR

Total RNA from 6 well plate transfections was extracted with TRIzol Reagent® (Ambion) according to manufacturer’s protocol and followed by precipitation with isopropanol. 1ug total RNA were treated with RQ1 RNase-Free DNase (Promega). 100ng DNased RNA was reverse transcribed with random hexamer (IDT) and Superscript III (Thermo Fischer). Quantitative Real-time PCR (RT-qPCR) was performed in 12μl Reactions using the PowerUp SYBR Green Master Mix (Thermo Fisher). For each construct RT-qPCR was performed with two sets of primers – complementary to Rluc and Fluc encoding sequence, respectively. RNA fold changes of test constructs were calculated with the 2 ^-ΔΔCt^ method and normalized to beta-Actin and Vimentin mRNA levels. The primers used are listed in Extended Data File 1.

### Statistical analysis

Box plots were generated with a web tool BoxPlotR. Box plots elements: centre lines show the medians; box limits indicate the 25th and 75th percentiles as determined by R software; whiskers extend 1.5 times the interquartile range from the 25th and 75th percentiles, outliers are represented by dots. n = 12 sample points. 2-tailed, paired samples t-test was performed in excel (version 2016) on samples as indicated.

## Results and discussion

### Inhibitory effect of *OPRL1* stop codon context in EMCV IRES vector

We further explored the effects of test sequences placed downstream of a reporter stop codon in an RT context in the bicistronic vector. The test sequences were: *AMD1 tail* as described in our previous work ( Yordanova *et al*., 2018) ( Figure 1A), a fragment of *ACTB* coding sequence of equivalent length (381 nt), and mouse ornithine decarboxylase 1 *(ODC1)* C-terminal PEST encoding region (492 nt). *ACTB* was selected to represent a neutral sequence optimized for efficient translation that is not expected to affect the reporter levels. *ODC1* PEST codes for a degradation signal ( Loetscher *et al*., 1991) and was selected to control for the effects that a degron could have on the reporter when placed downstream of an RT context. In agreement with our previous work, high-level readthrough of *AMD1 tail* resulted in a significant drop in reporter levels in a construct where *AMD1 tail* is placed downstream of the Rluc stop codon in the *OPRL1* RT context ( Figure 1B, compare *UGA* with *OPRL1*). We have attributed this effect to the inhibition of translation by ribosome stalling in *AMD1 tail* ( Yordanova *et al*., 2018). However, when we replaced *AMD1 tail* with the neutral *ACTB* (no decrease in reporter expected) or the *ODC1* PEST (<20% decrease expected) sequences, they exhibited similar inhibitory behaviour ( Figure 1B, compare UGA and *OPRL1*).

One possible explanation for the observed inhibition of reporter levels is that it results from the addition of *OPRL1* context sequence at the end of the reporter’s ORF. Both 5’ and 3’ nucleotides of *OPRL1* context contribute to RT efficiency. To explore the role of the 5’ *OPRL1* context, we tested constructs wherein ribosome access to the sequence beyond the stop codon is prevented. For this we substituted UGA in the *OPRL1* context with two UAA codons ( Figure 1B, *OPRL1* UAA). In addition, we tested constructs wherein *OPRL1* 5’ signal was truncated to only its two last codons instead of six ( Figure 1B, tr *OPRL1*). We have recently determined that just two codons 5’ of the *OPRL1* stop signal are sufficient for maximal readthrough (Loughran *et al* in preparation).

The termination products of *OPRL1* and *OPRL1* UAA constructs have the exact same amino acid composition, with both having the six *OPRL1* derived amino acids at their C-termini. For the *OPRL1* UAA construct the Rluc reporter levels are indicative of the availability/activity of the termination product only, while for the UGA construct both the RT product and the termination product contribute to the reporter levels. *OPRL1* UAA constructs exhibited very similar reporter levels as the *OPRL1* WT constructs ( Figure 1B) suggesting that the Rluc observed reduction was due to the occurrence of the six *OPRL1* codons just upstream of the stop codon and did not depend on downstream translation. Nonetheless, it should be noted that there is a small difference in the reporter levels between *OPRL1* and *OPRL1* UAA which is most significant for *AMD1* (p=10 ^-15^, t-test), less significant for *ODC1* PEST (p=10 ^-8^, t-test), and even less significant for *ACTB* (p=10 ^-4^, t-test). This could be due either to reduced stability of the RT products in which case any *AMD1 tail* destabilisation effect must exceed that of *ODC1* PEST degron or else it could be due to *AMD1 tail* translation having an inhibitory effect on the reporter’s translation.

Shortening the 5’sequence of *OPRL1* context by deleting four of the six codons largely recovered reporter levels with all three test sequences ( Figure 1B, tr *OPRL1*) supporting the idea that these four codons contribute to the observed effect. Like with the full 5’ RT context in *OPRL1*, the truncated form in tr *OPRL1* constructs exhibited similar reporter levels with all three test sequences. These findings would appear to argue against an *AMD1 tail* specific inhibitory effect in the RT constructs that we reported in our original work ( Yordanova *et al*., 2018).

To investigate if *OPRL1* 5’ context was interfering with reporter activities or whether it affected the protein levels, we performed western blotting which showed that the amount of detectable reporter protein was significantly reduced in the presence of the *OPRL1* context for all three test sequences ( Figure 1E, compare UGA and tr *OPRL1* vs *OPRL1* and *OPRL1* UAA). This indicates that in these reporters the *OPRL1* 5’ context does not simply interfere with Rluc activity. As expected, RT product was detected only with *ACTB* constructs.

The reduction of Rluc levels observed in the presence of *OPRL1* context could be due to a protein destabilising effect of the *OPRL1* derived peptide at the reporter’s C-terminus. However, western blotting revealed that the amount of detectable reporter protein was significantly reduced in both the termination and the RT product (as seen for *ACTB* constructs, Figure 1E), which argues against a C-terminal degron activity. In addition, it has been shown recently that the Venezuelan equine encephalitis virus (VEEV) RT stop codon context promotes ribosome stalling and it has been proposed that such stalling could be a general feature of RT promoting sequences ( Lashkevich *et al*., 2020). If so, the observed reduction of reporters containing *OPRL1* context could be attributed to slow peptide release and/or reporter mRNA degradation upon activation of ribosome quality control (RQC) pathways ( Ikeuchi *et al*., 2018; Joazeiro, 2019).

While Rluc activities normalized over Fluc values report a very similar picture for all three sequences tested ( Figure 1B), this is not the case for the absolute values of these reporters. Addition of the last six codons of *OPRL1* to UAA reporters greatly reduced Rluc levels for *ACTB* and *ODC1* PEST but not for *AMD1* ( Figure 1C, *OPRL1* UAA). The most likely explanation for this is that any reduction in Rluc for the *AMD1* reporters was masked by the increased stability of its corresponding mRNA as can be judged from its Fluc activity ( Figure 1D). With the exception of *AMD1* extensions, stop codon contexts that supported efficient termination (as in UGA and *OPRL1* UAA) had three-fold lower Fluc levels compared to those promoting RT ( *OPRL1* and tr *OPRL1*) which might be expected due to mRNA decay pathways such as Nonsense Mediated Decay (NMD) or No Go Decay (NGD) ( Shoemaker & Green, 2012) sensing reduced translation. For *AMD1,* enabling RT with *OPRL1* context did not lead to further stabilization of mRNA ( Figure 1D, *OPRL1* vs *OPRL1* UAA) which might be explained with the inhibitory effect on RNA levels that was reported to occur with *AMD1 tail* translation ( Yordanova *et al*., 2018; Wangen & Green, 2020).

**Figure 2.**
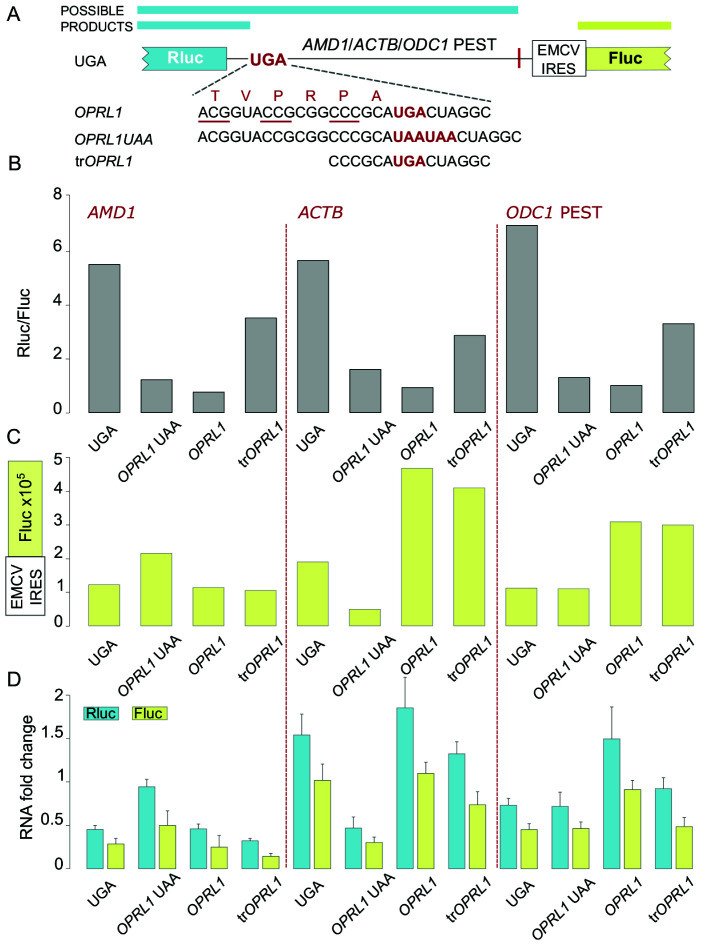
Fluc expression via the EMCV IRES is an accurate proxy of mRNA levels. ( **A**) Schematic of the *AMD1*, *ACTB* and *ODC1* PEST constructs where Rluc stop codon context is varied and Fluc expression is governed by the EMCV IRES. ( **B**) Normalized (Rluc/Fluc) activities. ( **C**) Absolute Fluc values. ( **D**) RNA levels change in folds as measured by qRT-PCR with two primer pairs targeting Rluc or Fluc.

Because Fluc expression levels are an indirect measurement of mRNA stability, we performed RT-qPCR to probe if Fluc expression levels reliably reflect mRNA levels ( Figure 2 and Extended Data Figure 1). We used two primer pairs that target Rluc or Fluc encoding sequences respectively. The results from the RT-qPCR supported the observations derived from the dual luciferase assay and confirmed that EMCV IRES driven Fluc expression levels are an accurate proxy of mRNA levels.

The relatively high Fluc expression levels observed when *AMD1 tail* is placed specifically downstream of *OPRL1* UAA context is intriguing and will need to be investigated further as it may shed light onto the properties of *AMD1 tail* and its function in the regulation of *AMD1* expression.

### Upstream reporter reduction depends on the RT promoting context and its efficiency

**Figure 3.**
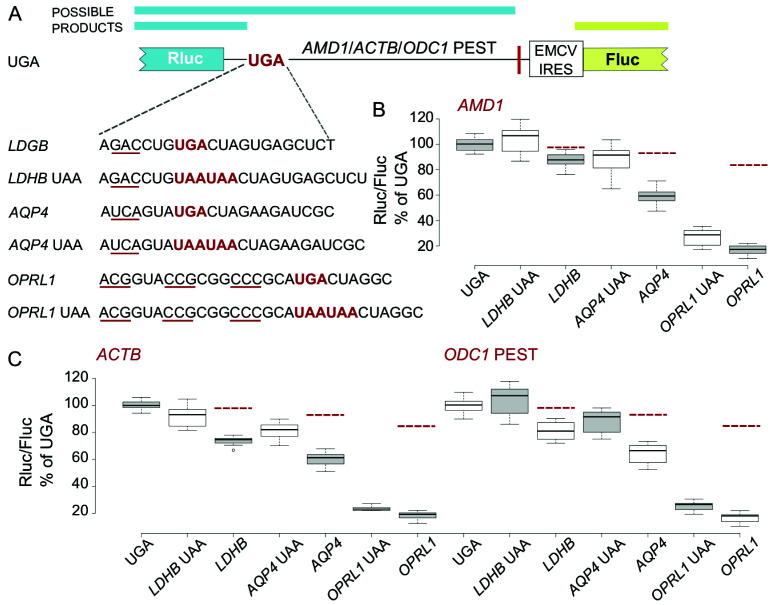
Effect of *LDHB*, *AQP4* and *OPRL1* RT contexts on Rluc levels in EMCV IRES vector. ( **A**) Schematic of the *AMD1*, *ACTB* and *ODC1* PEST constructs where Rluc stop codon context is varied and Fluc is expressed by EMCV IRES. ( **B**– **D**) Normalized (Rluc/Fluc) activities were calculated for *AMD1*, *ACTB* and *ODC1* PEST constructs as a percentage of the corresponding UGA construct. Red dashed lines indicate expected reporter levels in case that RT products are degraded. See Methods for box plot elements.

To determine whether these observations are specific to the *OPRL1* stop codon context we next tested the other two RT promoting contexts from our previous study ( Yordanova *et al*., 2018) ( Figure 3A). As already reported for the *AMD1 tail* reporters, gradually increasing RT efficiency with *LDHB*, *AQP4* and *OPRL1* resulted in disproportionate reductions in reporter levels ( Figure 3B). However, similar to the *OPRL1* RT context, constructs with *LDHB* and *AQP4* contexts exhibited the same trend when *AMD1 tail* was substituted with *ACTB* and *ODC1* PEST ( Figure 3C). With all three RT promoting contexts, a certain degree of recovery of reporter levels was observed upon substitution of UGA with UAAUAA. These results suggest that the observed reporter reduction depends on both, the RT context upstream of the stop codon as well as the RT efficiency but not on the extension sequence downstream of the stop codon.

### Uncoupling of *OPRL1* RT context and *AMD1* tail translation effects on reporter levels in a monocistronic vector

The results described so far argue that the changes in Rluc reporter levels align with RT context independently of translation downstream of the test sequence. These findings were unexpected because earlier studies with *OPRL1* RT context did not provide evidence for such effects on reporter levels which were found to be not substantially different in the presence or absence of *OPRL1* context ( Loughran *et al*., 2017). The main difference in the experimental approach in the 2017 study compared to our current analysis is the absence of the EMCV IRES.

**Figure 4.**
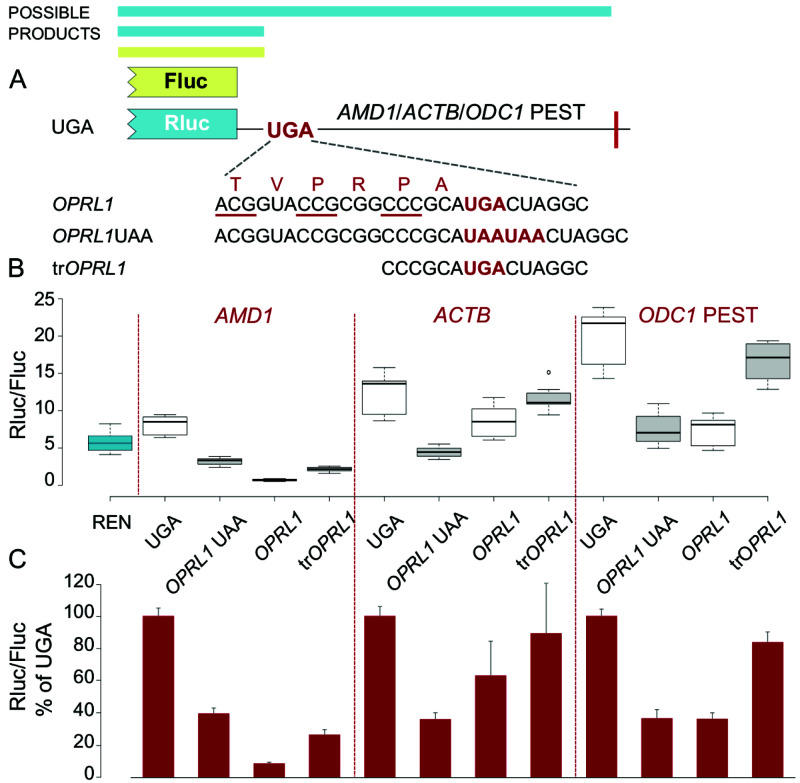
Effect of *OPRL1* RT context on Rluc levels in a vector without an IRES. ( **A**) Schematic of the *AMD1*, *ACTB* and *ODC1* PEST constructs where Rluc stop codon context is varied. Fluc is expressed by a separate vector. ( **B**) Normalized (Rluc/Fluc) luciferase activities. The leftmost blue box represents reporter levels with the empty Rluc expressing vector. ( **C**) Normalized (Rluc/Fluc) activities were calculated for the constructs from ( **B**) as percentages of the corresponding UGA construct. See Methods for box plot elements.

Therefore, to clarify these contradicting observations, we next tested the RT sequences shown in Figure 1A in a monocistronic vector that encodes Rluc and has no EMCV IRES ( Figure 4A). Cells were co-transfected with these Rluc constructs together with Fluc reporters expressed from a separate vector to control for transfection efficiencies. Because Fluc is expressed from a separate vector in this setup we do not account for RNA stability levels.

In the absence of EMCV IRES, the *OPRL1* UAA context resulted in similar levels of reporters with *AMD1*, *ACTB* and *ODC1* PEST ( Figure 4B and 4C, *OPRL1* UAA). Albeit milder, the reduction of reporter levels compared to UGA constructs, is consistent with the *OPRL1* context destabilising effect as revealed in the EMCV IRES vector ( Figure 1); While the *OPRL1* context resulted in similar to OPRL1 UAA reduction of reporter levels with *ACTB* and *ODC1* PEST, with *AMD1* construct it resulted in more than 10-fold reduction of reporter levels consistent with the reported inhibitory effect of *AMD1 tail* translation. Furthermore, with the trOPRL1 only a mild reduction in reporter levels was observed with *ACTB* and *ODC1* PEST compared to that with AMD1. 

These results suggest that the observed effects are related to the nature of the translated sequence downstream of the stop codon in the RT context. Critically, for *AMD1* the reduction of Rluc activity in *OPRL1* RT in comparison with the *OPRL1* UAA construct greatly exceed what would be expected if this was due to degradation of the RT product only, supporting our earlier claim ( Yordanova *et al*., 2018).

Reporter expression levels from the empty Rluc vector were in the range of those from *OPRL1* and *OPRL1* UAA constructs for *ACTB* and *ODC1* PEST. This is consistent with the previous study, which showed no change in reporter expression levels in the presence of *OPRL1* context ( Loughran *et al*., 2017) ( Figure 4B).

## Conclusions

In our investigation of ribosome stalling following stop codon readthrough in the human *AMD1* gene, we proposed a ribosome queuing model to explain downregulation of reporter genes fused with *AMD1 tail* ( Yordanova *et al*., 2018). To test the model, we varied readthrough context at the *AMD1* stop codon and observed disproportionately high inhibition of upstream reporters in response to increased readthrough efficiency as predicted by the model. Here we report that the observed reduction of upstream reporter levels is due to the RT context rather than due to *AMD1 tail* translation, contrary to our initial interpretations of the experiments presented in Figure 3C of the original publication. We also found that this inhibition is observed only in the reporter vector where the downstream reporter is under the control of an EMCV IRES.

This result helped us to uncouple the inhibitory effects of RT contexts and *AMD1 tail* translation on reporter’s expression. In a vector not using EMCV IRES initiation, reporter expression is reduced further when *AMD1 tail* is translated (due to readthrough). This reduction is not observed when *AMD1 tail* is replaced with unrelated sequences supporting our original claim that translation of *AMD1 tail* has an inhibitory effect on expression of upstream ORFs.

While the nature of molecular mechanisms responsible for the reported effects remains to be elucidated, our work extends the list of unexpected properties of IRES elements ( Payne *et al*., 2013; Shikama *et al*., 2010) and thus reinforces the need for caution in interpretation of data obtained with IRES containing reporters.

## Data availability

### Underlying data

Figshare: Stop codon readthrough contexts influence reporter expression differentially depending on the presence of an IRES. https://doi.org/10.6084/m9.figshare.16671103 ( Yordanova *et al*., 2021a).

This project contains the following underlying data:

- dataFigure_1.csv (Raw data dual luciferase assay for Figure 1.)
- dataFigure_2.csv (Raw data RT-qPCR and luciferase assay for Figure 2.)
- dataFigure_3.csv (Raw data dual luciferase assay for Figure 3.)
- dataFigure_4.csv (Raw data dual luciferase assay for Figure 4.)
- western_700. (Original unannotated western blot image.)

### Extended data

Figshare: Stop codon readthrough contexts influence reporter expression differentially depending on the presence of an IRES. https://doi.org/10.6084/m9.figshare.16676671 ( Yordanova *et al*., 2021b). 

This project contains the following extended data:

- Extended_Data_Figure_1.eps
- Extended_Data_Figure_2.eps
- Extended_Data_File_1.csv. (List of test sequences and primers.)

Data are available under the terms of the Creative Commons Attribution 4.0 license (CC BY 4.0).
