# Supplementary material for: Stop codon readthrough contexts influence reporter expression differentially depending on the presence of an IRES
Source: Wellcome Open Res. 2022 Jan 31;5:221. Originally published 2020 Sep 22. [Version 3] doi: 10.12688/wellcomeopenres.16231.3 (PMC8831848; doi:10.12688/wellcomeopenres.16231.3)
Supplement: Peer review history

# Peer review: Reviewer response for version 3

**DOI:** 10.21956/wellcomeopenres.19516.r48363

**Published:** 2022-02-10

**Authors:** Leos Shivaya Valasek

**Affiliations:** 1 Laboratory of Regulation of Gene Expression, Institute of Microbiology ASCR, Prague, Czech Republic

I want to thank the authors for addressing my comments. The impact of this article remains, in my opinion, partly debatable, but I agree that the red flag it raises is worth sharing with the community.

Is the work clearly and accurately presented and does it cite the current literature?

Partly

If applicable, is the statistical analysis and its interpretation appropriate?

I cannot comment. A qualified statistician is required.

Are all the source data underlying the results available to ensure full reproducibility?

Yes

Is the study design appropriate and is the work technically sound?

Yes

Are the conclusions drawn adequately supported by the results?

Partly

Are sufficient details of methods and analysis provided to allow replication by others?

Yes

Reviewer Expertise:

regulation of gene expression, translation initiation and termination, reinitiation, readthrough

I confirm that I have read this submission and believe that I have an appropriate level of expertise to confirm that it is of an acceptable scientific standard.

# Peer review: Reviewer response for version 2

**DOI:** 10.21956/wellcomeopenres.18736.r46372

**Published:** 2021-11-09

**Authors:** Eric Jan, Yihang Chen

**Affiliations:** 1 Department of Biochemistry and Molecular Biology, University of British Columbia, Vancouver, BC, Canada; 2 University of British Columbia, Vancouver, BC, Canada

The manuscript is continuation of the group's (Nature 2018) findings that translation of a stop codon readthrough tail in AMD1 results in inhibition of translation of the upstream reporter via a ribosome queuing mechanism. Here, using a dual luciferase reporter, they find that in reporters with a downstream EMCV IRES-FLuc, the inhibition of the upstream RLuc reporter is not dependent on the readthrough translation tail but dependent on the stop codon context of the C-term AMD1. However, using a monocistronic RNA without the EMCV IRES, they were able to observe inhibition of the upstream reporter that was dependent on the stop codon readthrough AMD1 tail ORF, in line with their original findings.

1. Unfortunately, the study is incomplete as there is not an explanation of why inclusion of the IRES affects the inhibitory effects of the upstream RLuc. In sum, the report leaves us hanging with several observations with no clear model or mechanistic insight. Follow-up experiments such as use of different IRESs may be provide insights or a mutant EMCV IRES or addressing whether there is competition between translation of the distinct ORFs? As it is, the interpretation that inclusion of the EMCV IRES is leading to these effects is not clear and there could be other models at play. As the authors state "findings provide little new information" to this mechanism but does clarify and forewarns the use of IRES- vs non-IRES containing reporters when studying this system. As the authors state, the mechanism underlying the effects of the IRES needs to be investigated further. However, I believe that this study has some merit with regards to getting this data out to other researchers as a forewarning as the choice of reporter systems is critical in studying this ribosome queuing mechanism properly. This may be in line with the scope and criteria of this publication format.
2. Figure 2D, why are there discrepancies (rather large) in the RNA levels by qRT-PCR of the RLuc vs FLuc if this was a single RNA produced? This begs the question whether inclusion of the EMCV IRES is somehow leading to cryptic splicing or cryptic promoter activity that complicates the interpretation of the RLuc and FLuc measurements, especially the RLuc/FLuc ratio. This important control needs to be interpreted carefully.
  Is this effect observed using transfection of reporter RNAs instead of DNA transfections? Transfection with reporter RNAs may be a more direct approach in studying this system.
  I would suggest to rewrite the section that the EMCV-FLuc is a proxy measurement of RNA levels (i.e. delete). The qRT-PCR assay measures the RNA levels directly.
3. I found some places of the manuscript a bit difficult to read in particular in the descriptions of Figures 2 and 3. Most of Figure 2 appears to be a replicate of Figure 1 (?).

Is the work clearly and accurately presented and does it cite the current literature?

Yes

If applicable, is the statistical analysis and its interpretation appropriate?

Partly

Are all the source data underlying the results available to ensure full reproducibility?

Yes

Is the study design appropriate and is the work technically sound?

Partly

Are the conclusions drawn adequately supported by the results?

Partly

Are sufficient details of methods and analysis provided to allow replication by others?

Yes

Reviewer Expertise:

My research area is on IRES mechanisms and translational controls. We have extensive experience with dual reporter assays containing IRESs.

We confirm that we have read this submission and believe that we have an appropriate level of expertise to confirm that it is of an acceptable scientific standard, however we have significant reservations, as outlined above.

# Response

**Published:** 2022-01-20

**Authors:** Pavel Baranov

**Affiliations:** Department of Biochemistry, University College Cork, Ireland

***Comment:***

*The manuscript is continuation of the group's (Nature 2018) findings that translation of a stop codon readthrough tail in AMD1 results in inhibition of translation of the upstream reporter via a ribosome queuing mechanism. Here, using a dual luciferase reporter, they find that in reporters with a downstream EMCV IRES-FLuc, the inhibition of the upstream RLuc reporter is not dependent on the readthrough translation tail but dependent on the stop codon context of the C-term AMD1. However, using a monocistronic RNA without the EMCV IRES, they were able to observe inhibition of the upstream reporter that was dependent on the stop codon readthrough AMD1 tail ORF, in line with their original findings.*

*Unfortunately, the study is incomplete as there is not an explanation of why inclusion of the IRES affects the inhibitory effects of the upstream RLuc. In sum, the report leaves us hanging with several observations with no clear model or mechanistic insight. Follow-up experiments such as use of different IRESs may be provide insights or a mutant EMCV IRES or addressing whether there is competition between translation of the distinct ORFs? As it is, the interpretation that inclusion of the EMCV IRES is leading to these effects is not clear and there could be other models at play. As the authors state "findings provide little new information" to this mechanism but does clarify and forewarns the use of IRES- vs non-IRES containing reporters when studying this system. As the authors state, the mechanism underlying the effects of the IRES needs to be investigated further. However, I believe that this study has some merit with regards to getting this data out to other researchers as a forewarning as the choice of reporter systems is critical in studying this ribosome queuing mechanism properly. This may be in line with the scope and criteria of this publication format.*

**Response:**

 We thank the reviewer and wish to emphasise that the reviewer is correct in that our main aim in this report is to forewarn readers of this IRES-reporter artefact and to reassure readers that our original conclusions are reproduced using monocistronic reporters. We concede that we cannot provide an explanation for the IRES artefact (beyond tentative speculative suggestions above) at this time, but we believe that this shouldn’t deter us from making this information publicly available.

***Comment:***

*Figure 2D, why are there discrepancies (rather large) in the RNA levels by qRT-PCR of the RLuc vs FLuc if this was a single RNA produced? This begs the question whether inclusion of the EMCV IRES is somehow leading to cryptic splicing or cryptic promoter activity that complicates the interpretation of the RLuc and FLuc measurements, especially the RLuc/FLuc ratio. This important control needs to be interpreted carefully.*

**Response:**

 The relative difference in RT-qPCR data when measuring RNA levels using two sets of primers (one that targets Rluc encoding sequence and the other targeting Fluc encoding sequence) is similar for all 12 constructs. This can be seen when normalising Fluc and Rluc RT-qPCR data for each construct to the UGA construct in each group of constructs (AMD1, PEST, ACTB) in Extended Data Figure 3. We suspect that the absolute differences between RT-qPCR measurements for the same constructs are due to differential primer binding efficiencies. 

***Comment:***

*Is this effect observed using transfection of reporter RNAs instead of DNA transfections? Transfection with reporter RNAs may be a more direct approach in studying this system.*

**Response:**

 We agree that RNA transfections would be a more direct way to study this system but one of our main aims is, as you pointed out, to forewarn readers about possible artefacts with dicistronic vectors employing IRESs. However, we have added a sentence that suggests that RNA transfections would be a useful alternative here.

***Comment:***

*I would suggest to rewrite the section that the EMCV-FLuc is a proxy measurement of RNA levels (i.e. delete). The qRT-PCR assay measures the RNA levels directly.*

**Response:**

 We thank the reviewer for pointing out this. What we intended to communicate was that RT-qPCR data confirmed that the Fluc levels (as measured by luciferase assay) are an accurate proxy of RNA levels. We have now clarified this in the text.

***Comment:***

*I found some places of the manuscript a bit difficult to read in particular in the descriptions of Figures 2 and 3. Most of Figure 2 appears to be a replicate of Figure 1 (?).*

**Response:**

 Figure 1 (now Figure 2) shows Luciferase assay data and western blotting of the products of expression of the 12 IRES containing constructs. Figure 2 (now Figure 3) presents RT-qPCR data from the same 12 IRES containing constructs.

# Peer review: Reviewer response for version 2

**DOI:** 10.21956/wellcomeopenres.18736.r46290

**Published:** 2021-11-02

**Authors:** Adam Geballe

**Affiliations:** 1 Division of Human Biology, Fred Hutchinson Cancer Research Center, Seattle, WA, USA

Yordanova and co-workers have revised their report in which they investigate the influence of translation termination vs. readthrough and post-termination protein coding regions on expression of the upstream reading frame, in both dicistronic and monocistronic reporter systems. The authors have done a large number of experiments and collected some intriguing data. Unfortunately, the data remain confusing and they do not, in my opinion, support any clear model or conclusions about what is the cause of the variation in expression patterns.

Is the work clearly and accurately presented and does it cite the current literature?

No

If applicable, is the statistical analysis and its interpretation appropriate?

I cannot comment. A qualified statistician is required.

Are all the source data underlying the results available to ensure full reproducibility?

Yes

Is the study design appropriate and is the work technically sound?

Partly

Are the conclusions drawn adequately supported by the results?

Partly

Are sufficient details of methods and analysis provided to allow replication by others?

Yes

Reviewer Expertise:

NA

I confirm that I have read this submission and believe that I have an appropriate level of expertise to state that I do not consider it to be of an acceptable scientific standard, for reasons outlined above.

# Response

**Published:** 2022-01-20

**Authors:** Pavel Baranov

**Affiliations:** Department of Biochemistry, University College Cork, Ireland

Reviewers 3 and 4 provided specific comments and suggestions which we addressed now and based on these we made significant changes to the manuscript. We believe that these are an improvement and hope that the Reviewer will find the clarity of the manuscript more satisfactory.

# Peer review: Reviewer response for version 2

**DOI:** 10.21956/wellcomeopenres.18736.r46410

**Published:** 2021-10-25

**Authors:** Leos Shivaya Valasek

**Affiliations:** 1 Laboratory of Regulation of Gene Expression, Institute of Microbiology ASCR, Prague, Czech Republic

The paper presented by Yordanova *et al.* builds on the previous findings by this group showing that the genuine stop codon of *AMD1 *mRNA is read through towards the next in-frame stop producing the AMD1 tail, where ribosomes stall, invoking ribosome queueing, which in turn inhibits readthrough. If the stall is prolonged, it severely suppresses AMD1 translation per se (Yordanova *et al.*, Nature, 2018). To test the general effect of this specific stalling/queueing *AMD1 *tail sequences in the referred paper, the authors used the Rluc reporter and placed its stop codon in the context of various readthrough permissive sequences, followed by the AMD1 tail, in a dual reporter vector with downstream reporter expression driven by the EMCV IRES. Doing so, they observed a striking disproportional reduction of upstream reporter activity in response to increased readthrough levels. As far as I understood it, and, frankly, it is not a “super-easy-to-follow” manuscript, the current study was aimed at understanding this disproportional reduction. Unexpectedly, the disproportionate reduction of upstream reporter activity was found not to be specific to the *AMD1 *tail, as might have been thought. It seems to occur as long as the readthrough stop codon context is present at the end of the reporter’s ORF, and is greatly influenced by the presence of EMCV IRES. Importantly, the monocistronic vector without an IRES confirmed the inhibitory effect of the *AMD1 *tail, as reported earlier.

 My honest impression is that this study was designed to serve three main purposes:

1. To refute doubts on the major conclusion from the earlier paper;
2. To cast doubts on the key reporter used in it;
3. And to warn colleagues to be cautious when using a similar set-up.

If true, it has served them well, the real pity is that the entire analysis is very mysterious and no explanation has been given for the unexpected effect of the IRES and the readthrough contexts on the activity of the Rluc reporter.

 Below I list a few issues – in the order of their appearance in the text – that might deserve the authors' attention.

1. Fig. 1 could feature a simple schematic of the AMD1 mRNA depicting the main ORF plus its tail and all features that the authors refer to in the main text. Why do you call the 125-codon long sequence between the two stops an ORF, as opposed to a C-terminal extension? Does it have AUG right after the first AMD1 stop? Or AMD1 is shorter than 125 codons and what you call the AMD1 tail is regular AMD1 plus its C-term. extension, together accounting for 125 codons? I guess the latter is correct, nonetheless, it was confusing and the schematic would help.
2. Does this not: “We have recently determined that just two codons 5’ of the *OPRL1 *stop signal are sufficient for maximal readthrough (Loughran *et al *in preparation).” contradict the following: ”Shortening the 5’sequence of *OPRL1 *context by deleting four of the six codons largely recovered reporter levels with all three test sequences (Figure 1B, tr *OPRL1*) supporting the idea that these four codons contribute to the observed effect.”? In my understanding, just two codons immediately 5’ of the *OPRL1 *stop signal” were preserved in tr OPRL1 - or did you mean any two codons of the six?
3. “With the exception of *AMD1 *extensions, stop codon contexts that supported efficient termination (as in UGA and *OPRL1 *UAA) had three-fold lower Fluc levels compared to those promoting RT ( *OPRL1 *and tr *OPRL1*) which might be expected due to mRNA decay pathways such as Nonsense Mediated Decay (NMD) or No Go Decay (NGD) (Shoemaker & Green, 2012) sensing reduced translation.“ Is there a reason to think of these as PTCs triggering NMD? Any EJCs nearby? As for NGD, efficient termination should not trigger it, unless the ribosomes do not get recycled. What do you think?
4. Figure 2: I would think that the RT-qPCR data, which I trust a lot more, only partly agree with the second reporter, IRES, measurements. I would suggest to normalize all data in this manuscript to RT-qPCR measurements using the IRES only for the comparison in Figure 2. Perhaps it will become less puzzling?
5. Could stalling and IRES-driven initiation on the same construct interfere with each other – sterically/mechanistically – causing the observed disproportion in your data?
6. Figure 4: Fluc is driven by its standard promoter here? Have you normalized your Rluc measurements to the Rluc mRNA levels using RT-qPCR?   

Thank you for giving me the opportunity to review this article.

Is the work clearly and accurately presented and does it cite the current literature?

Partly

If applicable, is the statistical analysis and its interpretation appropriate?

I cannot comment. A qualified statistician is required.

Are all the source data underlying the results available to ensure full reproducibility?

Yes

Is the study design appropriate and is the work technically sound?

Yes

Are the conclusions drawn adequately supported by the results?

Partly

Are sufficient details of methods and analysis provided to allow replication by others?

Yes

Reviewer Expertise:

regulation of gene expression, translation initiation and termination, reinitiation, readthrough

I confirm that I have read this submission and believe that I have an appropriate level of expertise to confirm that it is of an acceptable scientific standard, however I have significant reservations, as outlined above.

# Response

**Published:** 2022-01-20

**Authors:** Pavel Baranov

**Affiliations:** Department of Biochemistry, University College Cork, Ireland

***Comment:***

*The paper presented by Yordanova et al. builds on the previous findings by this group showing that the genuine stop codon of AMD1 mRNA is read through towards the next in-frame stop producing the AMD1 tail, where ribosomes stall, invoking ribosome queueing, which in turn inhibits readthrough. If the stall is prolonged, it severely suppresses AMD1 translation per se (Yordanova et al., Nature, 2018). To test the general effect of this specific stalling/queueing AMD1 tail sequences in the referred paper, the authors used the Rluc reporter and placed its stop codon in the context of various readthrough permissive sequences, followed by the AMD1 tail, in a dual reporter vector with downstream reporter expression driven by the EMCV IRES. Doing so, they observed a striking disproportional reduction of upstream reporter activity in response to increased readthrough levels. As far as I understood it, and, frankly, it is not a “super-easy-to-follow” manuscript, the current study was aimed at understanding this disproportional reduction. Unexpectedly, the disproportionate reduction of upstream reporter activity was found not to be specific to the AMD1 tail, as might have been thought. It seems to occur as long as the readthrough stop codon context is present at the end of the reporter’s ORF, and is greatly influenced by the presence of EMCV IRES. Importantly, the monocistronic vector without an IRES confirmed the inhibitory effect of the AMD1 tail, as reported earlier. My honest impression is that this study was designed to serve three main purposes:*

*To refute doubts on the major conclusion from the earlier paper;*

*To cast doubts on the key reporter used in it;*

*And to warn colleagues to be cautious when using a similar set-up.*

*If true, it has served them well, the real pity is that the entire analysis is very mysterious and no explanation has been given for the unexpected effect of the IRES and the readthrough contexts on the activity of the Rluc reporter.*

**Response:**

 The reviewer’s conclusions are partially correct. This study was not ‘designed’ to refute any doubts about our original paper or the key reporter - our intention was to further understand the mechanism. However, this approach did uncover an artefact from the reporter system we used in our original study. Our main aim in this report is to alert readers of this IRES-reporter artefact and more importantly to recapitulate our original conclusions using monocistronic reporters. We concede that we cannot provide an explanation for the IRES-induced artefact at this time, but we believe that this shouldn’t deter us from making this information publicly available to alert other researchers of potential artefacts using IRESs for similar purposes.

 Also, in relation to the first sentence of the Review, here we are measuring/reporting the inhibition of the reporter translation rather than the inhibition of readthrough.

***Comment:***

*Below I list a few issues – in the order of their appearance in the text – that might deserve the authors' attention.*

*Fig. 1 could feature a simple schematic of the AMD1 mRNA depicting the main ORF plus its tail and all features that the authors refer to in the main text. Why do you call the 125-codon long sequence between the two stops an ORF, as opposed to a C-terminal extension? Does it have AUG right after the first AMD1 stop? Or AMD1 is shorter than 125 codons and what you call the AMD1 tail is regular AMD1 plus its C-term. extension, together accounting for 125 codons? I guess the latter is correct, nonetheless, it was confusing and the schematic would help.*

**Response:**

 We thank the reviewer for the suggestion to include a schematic indicating *AMD1 *CDS and *AMD1 tail*. We now include a new figure (Figure 1) showing translation of *AMD1* mRNA from aggregated ribosome profiling data. We have indicated *AMD1* mRNA features such as the coding region (CDS), *AMD1 tail*, and the regulatory uORF MAGDIS. Between the CDS stop codon and the next inframe stop codon there is a 125 codon long ORF (We adhere to one of the definitions for an ORF namely the sequence between two stop codons in the same reading frame (Sieber et al., 2018). Like in the original publication, here we refer to the nucleotide sequence of this ORF as *AMD1 tail* and we use C-terminal extension when we refer to the putative protein product of this ORF translation.

***Comment:***

*Does this not: “We have recently determined that just two codons 5’ of the OPRL1 stop signal are sufficient for maximal readthrough (Loughran et al in preparation).” contradict the following: ”Shortening the 5’sequence of OPRL1 context by deleting four of the six codons largely recovered reporter levels with all three test sequences (Figure 1B, tr OPRL1) supporting the idea that these four codons contribute to the observed effect.”? In my understanding, just two codons immediately 5’ of the OPRL1 stop signal” were preserved in tr OPRL1 - or did you mean any two codons of the six?*

**Response:**

 The reviewer is correct in that in trOPRL1 only two codons are preserved immediately 5’ of the OPRL1 stop codon as indicated in the schematic. These were indeed sufficient for maximal readthrough (Loughran et al under review). Here we show that the inhibitory effect on Rluc levels does not depend on readthrough. This was confirmed by the constructs with efficient stop codons (OPRL1 UAA) that exhibited similar reduction of Rluc levels (in the absence of readthrough) as those with efficient readthrough. The inhibitory effect was relieved (albeit not fully removed) by the deletion of the first 4 of the 6 OPRL1 codons 5’ to the UGA codon. We now changed ‘the observed effect’ in the above sentence to ‘the observed inhibition’ to make it less confusing.

***Comment:***

*“With the exception of AMD1 extensions, stop codon contexts that supported efficient termination (as in UGA and OPRL1 UAA) had three-fold lower Fluc levels compared to those promoting RT (OPRL1 and tr OPRL1) which might be expected due to mRNA decay pathways such as Nonsense Mediated Decay (NMD) or No Go Decay (NGD) (Shoemaker & Green, 2012) sensing reduced translation.“ Is there a reason to think of these as PTCs triggering NMD? Any EJCs nearby? As for NGD, efficient termination should not trigger it, unless the ribosomes do not get recycled. What do you think?*

**Response:**

 The reviewer is correct that there are no EJCs nearby however NMD has been shown to be triggered also in an EJC independent manner (Zinshteyn et al., 2021) by means of sensing long non translated regions downstream of stop codon. We intended to point out to the possibility that such mRNAs could be sensed by NMD or similar (perhaps not yet identified) mechanisms and targeted for degradation. We agree that NGD is unlikely, and we have updated the text accordingly.

***Comment:***

*Figure 2: I would think that the RT-qPCR data, which I trust a lot more, only partly agree with the second reporter, IRES, measurements. I would suggest normalizing all data in this manuscript to RT-qPCR measurements using the IRES only for the comparison in Figure 2. Perhaps it will become less puzzling?*

**Response:**

 We thank the Reviewer for this suggestion. We have updated Figure 2 (now Figure 3) and Extended Figure 1 to show the Rluc activities normalised by RNA levels as measured by RT-qPCR (performed separately with primers for Rluc and Fluc) (panel 3B). RT-qPCR data were generated at the stage of manuscript revision for the 12 IRES containing constructs only and therefore unfortunately we could not normalize all data in the manuscript. However, normalising with the RT-qPCR data (2B) (now 3B) confirmed the pattern observed in 1B (now 2B) where Rluc activities were normalised by those of Fluc therefore providing confidence that Fluc expression levels measured with the luciferase assay accurately measure RNA levels.

***Comment:***

*Could stalling and IRES-driven initiation on the same construct interfere with each other – sterically/mechanistically – causing the observed disproportion in your data?*

**Response:**

 Yes this is one possibility, another possibility is that the presence of an IRES may influence formation of mRNA close loop conformation depending on the context (e.g., the time the ribosome dwell at a stop codon) by altering the overall dynamics of polysome complex three-dimensional structure. Subsequently this could alter initiation rate of the first reporter. Exhaustively testing possibilities like this would require a large number of additional constructs and testing which will not guarantee a definitive answer. We prefer not to do this for this study but may pursue this question separately. We included the above speculation into the last paragraph of the manuscript.  

***Comment:***

*Figure 4: Fluc is driven by its standard promoter here? Have you normalized your Rluc measurements to the Rluc mRNA levels using RT-qPCR?*

**Response:**

 In figure 4 (now Figure 5) the Fluc expression is driven from a standard promoter and from a separate construct. We don’t have RT-qPCR data for this experiment.

# Peer review: Reviewer response for version 2

**DOI:** 10.21956/wellcomeopenres.18736.r46291

**Published:** 2021-10-22

**Authors:** David Bedwell

**Affiliations:** 1 Department of Biochemistry and Molecular Genetics, University of Alabama at Birmingham, Birmingham, AL, USA

The first version of this article was very difficult to follow, and I’m afraid that this revised version is not much better. The authors made a series of constructs to test the effect of various components on expression of the R-Luc reporter. They previously showed that translation of AMD1 tail suppresses upstream reporter expression and that increased readthrough at the reporter stop codon results in increased reporter suppression. In this study, they further show that an inhibitory effect in addition to the AMD1 tail effect occurs when an IRES is present.

 The authors state “While our findings provide little new information regarding the functional role of *AMD1* tail, they raise caution for the use of viral IRES elements in expression vectors for studying mechanisms of mRNA translation.” The caution about using IRES elements is valid, but I don’t think the overall new information presented here merits indexing.

 I previously concluded that this really doesn’t add much to our understanding of how readthrough may inhibit translation of an ORF. This conclusion still stands, so I am still unwilling to Approve this manuscript.

Is the work clearly and accurately presented and does it cite the current literature?

No

If applicable, is the statistical analysis and its interpretation appropriate?

Yes

Are all the source data underlying the results available to ensure full reproducibility?

Yes

Is the study design appropriate and is the work technically sound?

Partly

Are the conclusions drawn adequately supported by the results?

Partly

Are sufficient details of methods and analysis provided to allow replication by others?

Yes

Reviewer Expertise:

NA

I confirm that I have read this submission and believe that I have an appropriate level of expertise to confirm that it is of an acceptable scientific standard, however I have significant reservations, as outlined above.

# Peer review: Reviewer response for version 1

**DOI:** 10.21956/wellcomeopenres.17828.r40919

**Published:** 2020-10-30

**Authors:** David Bedwell

**Affiliations:** 1 Department of Biochemistry and Molecular Genetics, University of Alabama at Birmingham, Birmingham, AL, USA

This study sought to test the hypothesis that the severe suppression of reporters fused to *AMD1 tail* was due to stop codon readthrough in *AMD1* mRNA followed by ribosome stalling in the *AMD1 tail*. To do this, the authors placed the reporter stop codon in the context of readthrough permissive sequences in a dual reporter vector with downstream reporter expression driven by the EMCV IRES. Consistent with their hypothesis, they observed an inverse correlation between upstream reporter activity in response to increased readthrough levels. They also found that this inhibition is observed only in the reporter vector where the downstream reporter is under the control of an EMCV IRES.

 The authors concluded that the observed reduction of upstream reporter levels correlates with the RT context rather than to AMD1 tail translation, contrary to the hypothesis they derived from data they obtained in a prior publication. However, this study was not really satisfying at any level, and no strong evidence was presented to support most of the conclusions made. As a result, we are left with a correlative study that really doesn’t add much to our understanding of how read through may inhibit translation of an ORF.

Is the work clearly and accurately presented and does it cite the current literature?

No

If applicable, is the statistical analysis and its interpretation appropriate?

Yes

Are all the source data underlying the results available to ensure full reproducibility?

Yes

Is the study design appropriate and is the work technically sound?

Partly

Are the conclusions drawn adequately supported by the results?

Partly

Are sufficient details of methods and analysis provided to allow replication by others?

Yes

Reviewer Expertise:

I have worked on translation termination and stop codon read through for 25 years. As such, I think I am qualified to review this study.

I confirm that I have read this submission and believe that I have an appropriate level of expertise to state that I do not consider it to be of an acceptable scientific standard, for reasons outlined above.

# Response

**Published:** 2021-10-04

**Authors:** Pavel Baranov

**Affiliations:** Department of Biochemistry, University College Cork, Ireland

*Comment:*

*This study sought to test the hypothesis that the severe suppression of reporters fused to AMD1 tail was due to stop codon readthrough in AMD1 mRNA followed by ribosome stalling in the AMD1 tail.*

*To do this, the authors placed the reporter stop codon in the context of readthrough permissive sequences in a dual reporter vector with downstream reporter expression driven by the EMCV IRES. Consistent with their hypothesis, they observed an inverse correlation between upstream reporter activity in response to increased readthrough levels.*

*They also found that this inhibition is observed only in the reporter vector where the downstream reporter is under the control of an EMCV IRES.*

 Response:

 We apologise for insufficient clarity of our original manuscript, the Reviewer’s summary does not reflect the content of the manuscript. We have already shown in our original publication on AMD1 that translation of AMD1 tail results in suppression of upstream reporters and that increasing readthrough efficiency at the reporters stop codon results in increased reporter suppression. We have also demonstrated that ribosomes stall at the end of AMD1 tail.

 We have already used the readthrough permissive sequences in our AMD1 paper but as explained in the response to Reviewer 1 we aimed at using this approach for an expanded study of various stalling sequences. It was during the early stage of this work that we made the current observations.

 The statement “They also found that this inhibition is observed only in the reporter vector where the downstream reporter is under the control of an EMCV IRES.” is a key misunderstanding. The inhibitory effect non-specific to AMD1 tail was observed with the IRES vector while the AMD1 specific inhibition was observed with the monocistronic vector.  

*Comment:*

*The authors concluded that the observed reduction of upstream reporter levels correlates with the RT context rather than to AMD1 tail translation, contrary to the hypothesis they derived from data they obtained in a prior publication.*

 Response:

 In the IRES vector indeed reduction of upstream reporter levels correlates with the RT context, however in the monocistronic vector the reporter reduction is specific to AMD1 tail. Taken together these results are supportive of our conclusion published previously. However, our current observations also introduce new aspects specific to the IRES vector and unrelated to AMD1 tail.

*Comment:*

*However, this study was not really satisfying at any level, and no strong evidence was presented to support most of the conclusions made. As a result, we are left with a correlative study that really doesn’t add much to our understanding of how read through may inhibit translation of an ORF.*

 Response:

 Indeed, we fail to provide a mechanistic explanation of our findings. Due to the unusual nature of the observations it might not be straightforward to uncover the mechanism behind the differential effects in the two vectors. However, we think it is very important to present these observations as they will no doubt be of use to researchers using similar reporter systems or for anyone who will study the mechanism of AMD1 translation control that we reported earlier.

# Peer review: Reviewer response for version 1

**DOI:** 10.21956/wellcomeopenres.17828.r40566

**Published:** 2020-10-05

**Authors:** Adam Geballe

**Affiliations:** 1 Division of Human Biology, Fred Hutchinson Cancer Research Center, Seattle, WA, USA

Yordanova and colleagues report follow-up studies of their Nature paper in which they found that translational readthrough at the AMD1 stop codon, leading to translation of a C-terminal extension (AMD1 tail), inhibited translation of the coding region. The authors had proposed that the inhibitory mechanism was due ribosome stalling at the downstream stop codon and queuing of ribosomes on the mRNA.  A more recent paper using ribosome profiling from another group confirmed that ribosomes translate the AMD1 tail, but found no evidence to support ribosome queuing. To evaluate their model further, these authors now test the prediction that increasing readthrough past the stop codon should increase the queuing and potentiate the inhibitory effect of the AMD1 tail. Using dicistronic constructs, they found that increasing the readthrough at the end Rluc results in lower Rluc/Fluc expression, but the effect does not depend on the AMD1 tail nor does it even require that readthrough onto the tail. They then tested another set of stop codon elements that allow readthrough and found that a higher level of readthrough efficiency resulted in more inhibition, but again, the effect was not dependent on the AMD1 tail. However, when they tested these stop codon contexts and tail sequence in monocistronic constructs, they then found that the AMD1 tail may be important for inhibition.

 The data are interesting but unconvincing in support of any clear conclusions. The results do suggest that the impact of the dicistronic constructs does not depend on the AMD1 tail, and there are modest differences in the impacts of the AMD1 tail vs the other tails in the monocistronic constructs, but the other tails have intermediate effects. There are multiple uncertain assumptions and other findings that are not well explained.

**Specific comments:**

- Abstract: “Monocistronic reporters with the same readthrough context sequence exhibit only a modest reduction” is confusing since there is a strong inhibitory effect with the AMD1 tail in the OPRL1 monocistronic construct and the other two do inhibit moderately.
- The authors report as “personal communication” that the truncated ORFL1 stop codon is sufficient for maximal readthrough, but this finding is quite important for interpreting many of the results and so the data should be presented.
- The authors state that the LDHB, AQP4, and OPRL1 RT sequences cause 2.5%, 6%, and 17% readthrough, but the paper cited (Loughran et al 2014) reports the latter OPRL1 cause 31% readthrough. A later paper revised that number to 17%. Might these numbers vary depending on the precise construct and context of the experiment? If so, it could impact the interpretation of some of the data (e.g. in Figure 2). 
- Page 5.  The argument “against a C-terminal degron activity” mediated by the ORFL1-derived peptide is not convincing. The fact that both the termination and RT products are lower in the OPRL1 and ORPL1UAA constructs does not rule out the possibility that the ORFL1 peptide (more so than the truncated version) is a degron that acts to degrade the short and longer proteins. 
- In Fig. 1D, the variation in Fluc expression is hard to explain by any simple model. Among other confusing results, how do the authors explain the differing effects on Fluc expression of *trOPRL1* in the AMD1 construct vs. in the *ACTB *and *ODC1 PEST* constructs? It may be true that the EMCV IRES is unreliable as they propose, but it is not clear it is really the problem nor how it might be misleading. There could be effects on IRES structure and function or on mRNA degradation of the dicistronic RNAs that results from upstream ORF, but they don’t provide direct evidence for any model. The authors generally interpret changes in the Fluc levels as being measures of mRNA, but that may not be incorrect, even for the monocistronic constructs. Some additional data, such as measuring RNA levels might help evaluate these alternatives.  Alternatively, other methods might be needed.
- Interpretation of the UAA constructs depends on the UAAUAA always being a strong terminator, which seems likely, but is unproven. The UAAUAA does reduce expression of the larger proteins in Fig. 1E but what are the other bands in the immunoblot that appear larger than the main protein? Are they background artifacts or other readthrough products? 
- How many independent replicates of the transfections were done?  Presumably at least several, but given the vagaries of transfection assays, coupled with the possibility that the Fluc may not be a good internal control, it is important to know that the data presented are highly reproducible.

Is the work clearly and accurately presented and does it cite the current literature?

No

If applicable, is the statistical analysis and its interpretation appropriate?

I cannot comment. A qualified statistician is required.

Are all the source data underlying the results available to ensure full reproducibility?

Yes

Is the study design appropriate and is the work technically sound?

Partly

Are the conclusions drawn adequately supported by the results?

Partly

Are sufficient details of methods and analysis provided to allow replication by others?

Yes

Reviewer Expertise:

I have been studying protein synthesis regulatory mechanism for >30 years. I discovered and characterized the mechanism of one of the early examples of ribosomal stalling in eukaryotes and have remain interested in this area. I think I am quite well qualified to evaluate this report.

I confirm that I have read this submission and believe that I have an appropriate level of expertise to state that I do not consider it to be of an acceptable scientific standard, for reasons outlined above.

# Response

**Published:** 2021-10-04

**Authors:** Pavel Baranov

**Affiliations:** Department of Biochemistry, University College Cork, Ireland

*Comment:*

*Yordanova and colleagues report follow-up studies of their Nature paper in which they found that translational readthrough at the AMD1 stop codon, leading to translation of a C-terminal extension (AMD1 tail), inhibited translation of the coding region. The authors had proposed that the inhibitory mechanism was due ribosome stalling at the downstream stop codon and queuing of ribosomes on the mRNA.  A more recent paper using ribosome profiling from another group confirmed that ribosomes translate the AMD1 tail, but found no evidence to support ribosome queuing.*

*To evaluate their model further, these authors now test the prediction that increasing readthrough past the stop codon should increase the queuing and potentiate the inhibitory effect of the AMD1 tail. Using dicistronic constructs, they found that increasing the readthrough at the end Rluc results in lower Rluc/Fluc expression, but the effect does not depend on the AMD1 tail nor does it even require that readthrough onto the tail.*

*They then tested another set of stop codon elements that allow readthrough and found that a higher level of readthrough efficiency resulted in more inhibition, but again, the effect was not dependent on the AMD1 tail. However, when they tested these stop codon contexts and tail sequence in monocistronic constructs, they then found that the AMD1 tail may be important for inhibition.*

*The data are interesting but unconvincing in support of any clear conclusions. The results do suggest that the impact of the dicistronic constructs does not depend on the AMD1 tail, and there are modest differences in the impacts of the AMD1 tail vs the other tails in the monocistronic constructs, but the other tails have intermediate effects. There are multiple uncertain assumptions and other findings that are not well explained.*

 Response:

 We thank the reviewer for their comments which together with those of reviewer 2 suggest that we have failed to clearly communicate the aims of our study.

 This current study is not a further examination of AMD1 tail properties although the presented observations were made during such an investigation. The current study reveals the observation that some sequences fused 3’ to that encoding Rluc, affect this reporter levels differentially in a monocistronic vector and in a bicistronic vector wherein the second reporter Fluc is expressed via the EMCV IRES.

 The reviewer mentions that “To evaluate their model further, these authors now test the prediction that increasing readthrough past the stop codon should increase the queuing and potentiate the inhibitory effect of the AMD1 tail”.

 This is not so and we apologise for not expressing the aim of this report clearly. This experiment has been done already for our original publication on AMD1 using the three different readthrough (RT) promoting contexts featured in the current study too. There we did not test control sequences alongside AMD1 tail and, in hindsight, erroneously interpreted the results of enhanced reporter inhibition with increasing RT efficiencies as evidence for ribosome queuing in AMD1 tail.

 Based on these previously published experiments we thought that using readthrough enabled ribosome titration was a good approach to study the dynamics of ribosome stalling (and potential queuing) not only in AMD1 tail but in other verified or candidate stalling sequences. We therefore designed a comparative study where among other approaches we planned to use RT contexts to further examine AMD1 tail and other selected sequences. ACTB and ODC PEST were chosen to serve as control sequences where stalling was not reported. Early on within this work we made the unusual observation presented here.

 In this manuscript we report our finding that when AMD1 tail, ACTB and ODC PEST test sequences were fused 3’ to Rluc via an RT promoting context, they support similar inhibition of Rluc levels when tested in a dual luciferase vector where the second reporter Fluc is expressed via the EMCV IRES. This suggests that our interpretation of the experiments with three different stop codons contexts was incorrect in the original paper because the inhibition observed in figure 3d of our original study is not specific to AMD1 tail.

 However, when these same Renilla-RT context-test sequence cassettes were tested in a monocistronic vector without the EMCV IRES, the test sequences caused the expected differential effects on Rluc levels. These effects are consistent with the AMD1 tail translation-mediated inhibitory effects already described. Therefore, with respect to AMD1 tail properties, the current study does not add new information, nor does it alter our conclusions from the earlier paper.  

 We are interested in further investigating the mechanism responsible for the link observed here but this is not straightforward, and it might take a while to elucidate. However, we consider it important to present these findings for  other researchers who may be using similar experimental systems. Apart from being helpful for the avoidance of potential artefacts, the results could also be stimulatory for others to research the underlying mechanism.

 Prompted by the reviewer’s comments we amended the manuscript in order to describe our findings and the purpose of the manuscript more clearly. We have also performed more experiments including RT-qPCR to probe the accuracy of an IRES controlled expression of a reporter as a proxy for mRNA levels.

*Comment:*

*Specific comments:*

*Abstract: “Monocistronic reporters with the same readthrough context sequence exhibit only a modest reduction” is confusing since there is a strong inhibitory effect with the AMD1 tail in the OPRL1 monocistronic construct and the other two do inhibit moderately.*

 Response:

 Thank you for pointing this out, it was indeed not clearly described and we have now changed the abstract to address this. The AMD1 tail has a strong inhibitory effect in the monocistronic vector unlike ACTB and ODC PEST that have only a modest effect. This is unlike in the bicistronic IRES vector where all three test sequences have the same inhibitory effect.

*Comment:*

*The authors report as “personal communication” that the truncated ORFL1 stop codon is sufficient for maximal readthrough, but this finding is quite important for interpreting many of the results and so the data should be presented.*

 Response:

 This finding is a part of another study that focuses on investigation of OPRL1 readthrough context. However it is currently under preparation. We would like to avoid duplicate publication of the same data and therefore made it available through figshare (doi:10.6084/m9.figshare.16661023).

*Comment:*

*The authors state that the LDHB, AQP4, and OPRL1 RT sequences cause 2.5%, 6%, and 17% readthrough, but the paper cited (Loughran et al 2014) reports the latter OPRL1 cause 31% readthrough. A later paper revised that number to 17%. Might these numbers vary depending on the precise construct and context of the experiment? If so, it could impact the interpretation of some of the data (e.g. in Figure 2).*

 Response:

 Initially the OPRL1 readthrough promoting context was tested in a dual luciferase vector pDluc (with no IRES) where the RT product is a fusion of Rluc and Fluc and with this method the RT efficiency was shown to be 31% (Loughran et al 2014 (PMID: 25013167)).  

 Later, we developed a new vector (pSGDluc) that eliminates the reporter distortions caused by fused test sequences where the two luciferase reporters are flanked by two StopGo elements that prevent formation of an Rluc-Fluc fusion and the RT efficiency was calculated at 17% (Loughran et al 2017 (PMID: 28442579)). Western blotting and densitometry confirmed 17% readthrough for OPRL1 in both pDLuc and pSGDLuc.

 In both cases, RT efficiency is calculated from the Fluc/Rluc ratio of a readthrough construct normalised to the Fluc/Rluc ratio of an inframe control construct. The variation (17% and 31%) between the two vector systems was due to the fact that the inframe control from the earlier study had distorted Fluc/Rluc ratio likely due to the altered activity within the fusion product. 

 In addition, the OPRL1 UGA absolute Rluc values were not significantly different in pDluc, pSGDluc and RLuc expressed from a monocistronic vector (figure 2A from Loughran et al 2017 paper (PMID: 28442579)). This together with the current results from the monocistronic vector suggested that the differences we observed with the bicistronic vector might be the due to the presence of the EMCV IRES – a feature absent in the other vector systems (pDluc, pSGDluc and monocistronic).    

 We first cited the Loughran et al 2014 paper (PMID: 25013167) because this is where the three RT candidates were first experimentally verified. We now cite the Loughran et al 2017 paper (PMID: 28442579) as well.

*Comment:*

*Page 5.  The argument “against a C-terminal degron activity” mediated by the ORFL1-derived peptide is not convincing. The fact that both the termination and RT products are lower in the OPRL1 and ORPL1UAA constructs does not rule out the possibility that the ORFL1 peptide (more so than the truncated version) is a degron that acts to degrade the short and longer proteins.*

 Response:

 We agree, it is possible that OPRL1 derived peptide destabilizes the protein. However, we suspect that an Rluc fused degron would affect Rluc levels similarly in the IRES vector and the monocistronic vector used in this study as well as in the pDluc vector used in the previous Loughran et al. 2014 and 2017 studies. However, there is no indication that the OPRL1 signal acts as a degron in pDluc (figure 2A from Loughran et al 2017 (PMID: 28442579)).

*Comment:*

*In Fig. 1D, the variation in Fluc expression is hard to explain by any simple model. Among other confusing results, how do the authors explain the differing effects on Fluc expression of trOPRL1 in the AMD1 construct vs. in the ACTB and ODC1 PEST constructs? It may be true that the EMCV IRES is unreliable as they propose, but it is not clear it is really the problem nor how it might be misleading.*

 Response:

 We agree, unfortunately we have no simple explanatory model. The strikingly different Fluc behaviour in AMD1 construct is very surprising and unexpected. Therefore, we repeated the cloning of this construct independently to be sure that this is not an artifact of a mutation somewhere in the vector. We obtained the same result with an independently made construct. While we failed to explain this surprising observation, it shouldn’t deflect us from the main message of this manuscript that the inhibition observed is not specific to AMD1 tail.

*Comment:*

*There could be effects on IRES structure and function or on mRNA degradation of the dicistronic RNAs that results from upstream ORF, but they don’t provide direct evidence for any model. The authors generally interpret changes in the Fluc levels as being measures of mRNA, but that may not be incorrect, even for the monocistronic constructs. Some additional data, such as measuring RNA levels might help evaluate these alternatives. Alternatively, other methods might be needed.*

 Response:

 We agree with the reviewer that it is important to verify if IRES driven Fluc expression reliably reflects mRNA levels. To address this, we performed RT-qPCR for all twelve test constructs from Figure 1 with two sets of primers targeted to both Rluc and Fluc coding sequences (new Figure 2).

 For this experiment transfections were performed in 6 well plates with one well per construct. Part of the lysate was used for luciferase assay (Figure 2,B and C). Two independent experiments were performed on separate days. The results of one of these experiments are shown on Figure 2. The other experiment is available as Extended Data Figure 1.

 The Fluc activities observed with AMD1 and ACTB remained as observed before. Moreover, these were confirmed by the RT-qPCR experiments that showed very good correlation between IRES driven Fluc expression levels and mRNA levels as measured with RT-qPCR with both primers for Rluc and Fluc (Figure 2,D and Extended Data Figure 1,D). Based on this we can conclude that the IRES-driven absolute values of Fluc expression reliably reflect mRNA levels.

 With one of the replicate experiments, we did not observe the same Fluc activities as seen previously with ODC PEST, Extended Data Figure 1. It is important to emphasise that absolute luciferase values fluctuate due to variable transfection efficiencies which are very difficult to reliably control (only the ratio is reliable). However, within an individual experiment IRES-driven Fluc activities and RT-qPCR-determined FLuc levels are in a very good agreement.

*Comment:*

*Interpretation of the UAA constructs depends on the UAAUAA always being a strong terminator, which seems likely, but is unproven. The UAAUAA does reduce expression of the larger proteins in Fig. 1E but what are the other bands in the immunoblot that appear larger than the main protein? Are they background artifacts or other readthrough products?*

 Response:

 The heavier bands are unspecific with an exception of ACTB in OPRL1 contexts which are indeed likely readthrough products. They disappear in both UGA and UAAUAA constructs.

*Comment:*

*How many independent replicates of the transfections were done?  Presumably at least several, but given the vagaries of transfection assays, coupled with the possibility that the Fluc may not be a good internal control, it is important to know that the data presented are highly reproducible.*

 Response:

 Indeed, this is an important point, see above regarding reproducibility of absolute values. To increase our confidence in these data we cloned again from scratch the AMD1 IRES constructs and transformed again these together with the ACTB and ODC PEST constructs. In addition, we sequenced long stretches containing the EMCV IRES and full length Fluc to exclude the possibility that a mutation has occurred in these areas that is affecting differentially Fluc expression of AMD1 OPRL1 UAAUAA and we saw no evidence for this.

 The data for Figure 1 is from 12 independent transfections which were performed in the same day with cells that were split in three plates the day before. We have now repeated the transfections in 96 well plates several times and on different days and with cells at different number of passages (Extended Data Figure 2).
